# Supplementary material for: Austrian Raw-Milk Hard-Cheese Ripening Involves Successional Dynamics of Non-Inoculated Bacteria and Fungi
Source: Foods. 2020 Dec 11;9(12):1851. doi: 10.3390/foods9121851 (PMC7763656; doi:10.3390/foods9121851)

Figure S1. 1D dot-plots of 18S rRNA dPCR system. Examples of the representative results required for dMIQE (Supplementary Table) are shown. Rind cheese DNA samples A) without enzymatic digestion, B) with enzymatic digestion (EcoRI), C) effect of dilution and digestion of the B90 sample. Each dot represents one partition. For each sample, droplets are depicted according to the event (number of droplet as read during reading; x-axis) and its fluorescence (Blue/FAM; y-axis). Blue dots represent the positive droplets. Grey dots represent the negative droplets. No positive partition has been detected in the NTC.

a)

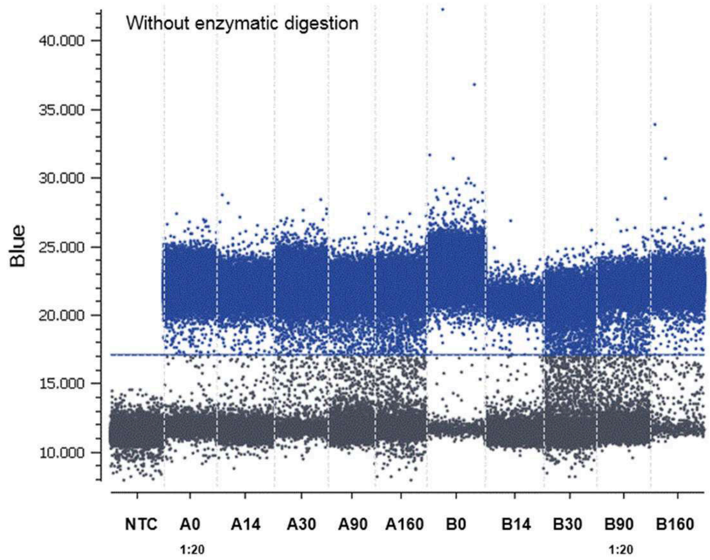

b)

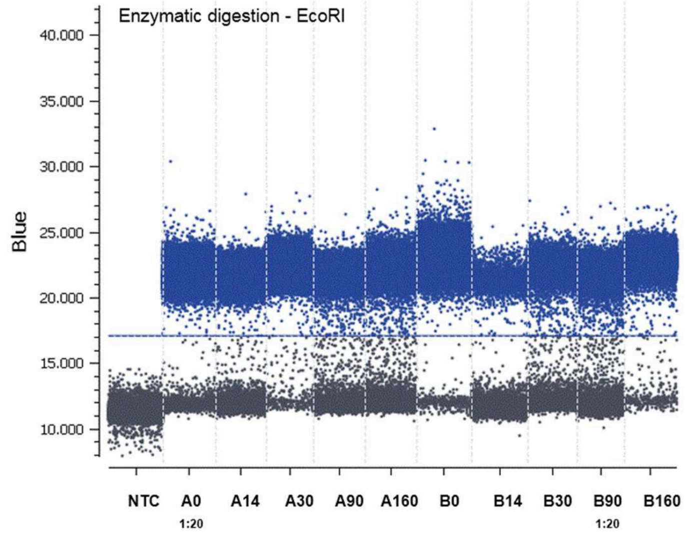

c)

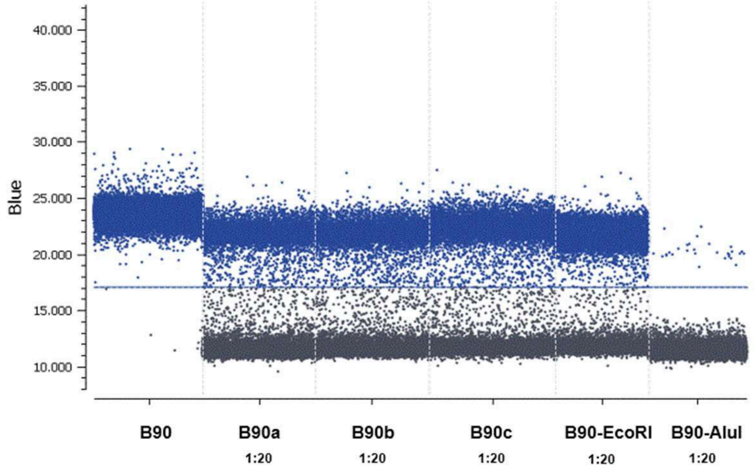

Supplement: Supplementary file 1 [file foods-09-01851-s001.zip › Figure_S1-examples_dPCR.pdf]
