# Supplementary material for: Austrian Raw-Milk Hard-Cheese Ripening Involves Successional Dynamics of Non-Inoculated Bacteria and Fungi
Source: Foods. 2020 Dec 11;9(12):1851. doi: 10.3390/foods9121851 (PMC7763656; doi:10.3390/foods9121851)

Figure S2. Comparison of qPCR and dPCR. qPCR fungal cell equivalents (FCEs) and dPCR stock concentration (Cp) per 0.5 g cheese rind during ripening in two different cheese production facilities are shown. A set of 200 cheese rind gDNA samples was pooled (n=10) according to their ripening time points (0, 14, 30, 90, and 160 days) and compared. To study the effect of DNA digestion (outside target-enzyme EcoRI) on the performance of the dPCR assay, quantification experiments were performed in parallel with non-digested and digested aliquots of the same DNAs. Coefficient of determination (R^2) values: qPCR vs dPCR without enzymatic digestion: facility A: R^2= 0.998; facility B: R^2=0.903. qPCR vs dPCR with enzymatic digestion: facility A: R^2= 0.998; facility B: R^2=0.893.

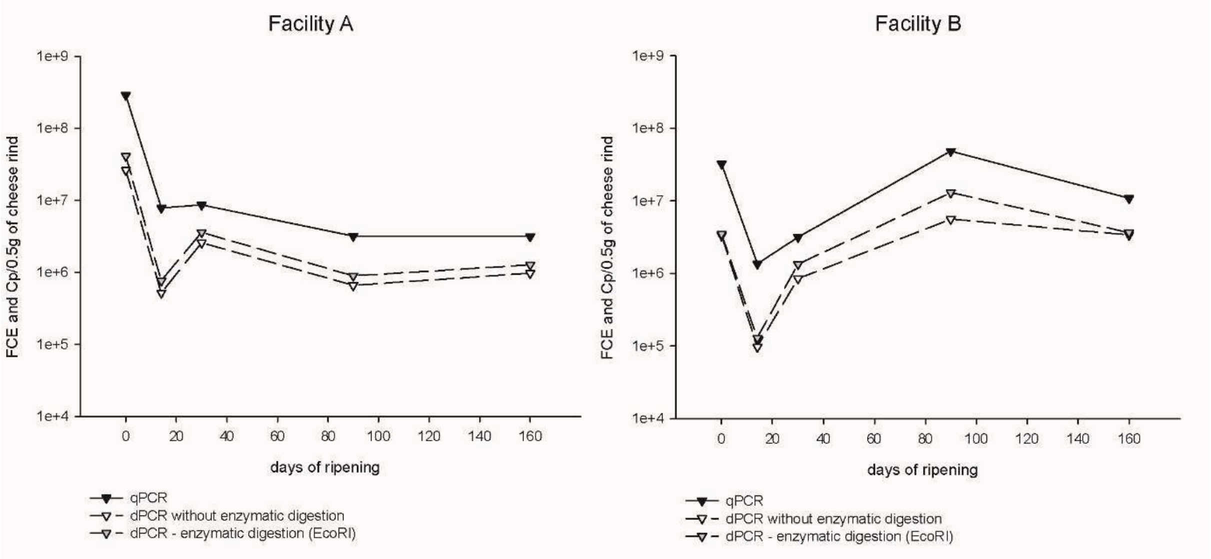

Supplement: Supplementary file 1 [file foods-09-01851-s001.zip › Figure_S2-qPCR_vs_dPCR.pdf]
