# Supplementary material for: Austrian Raw-Milk Hard-Cheese Ripening Involves Successional Dynamics of Non-Inoculated Bacteria and Fungi
Source: Foods. 2020 Dec 11;9(12):1851. doi: 10.3390/foods9121851 (PMC7763656; doi:10.3390/foods9121851)
Supplement: Supplementary file 1 [file foods-09-01851-s001.zip › Table_S1-MIQE_quidelines_qPCR.pdf]

Table S1. Minimum information for publication of quantitative real-time PCR experiments (MIQE guidelines qPCR) for 18S rRNA gene qPCR.

Item	Importance	Remarks
**Experimental design**		**18S/FungiQuant Assay**
Definition of experimental and control groups	E[1]	Two cheese diary facilities, A and B
Number within each group	E	Number of samples within each group (n=100), number of subgroups (n=5): ripening days 0; 14; 30; 90; 160
Assay carried out by core lab or investigator's lab?	D[2]	Investigator's lab
Acknowledgement of authors' contributions	D	See main manuscript
**Sample**		
Description	E	DNA isolated from cheese rinds
Volume/mass of sample processed	D	250 mg pellet of the homogenized cheese rind sample in duplicate
Microdissection or macrodissection	E	Not relevant, environmental material
Processing procedure	E	DNA isolation using PowerSoil™ DNA Isolation kit
If frozen - how and how quickly?	E	Samples were stored on ice during transport to the laboratory and processed immediately. After DNA isolation, samples were frozen within 10 minutes at -80°C.
If fixed - with what, how quickly?	E	Samples were not fixed
Sample storage conditions and duration	E	Cheese rind samples were processed immediately. DNAs were stored 1-6 months at -80°C, after thawing on ice, DNA samples were applied to the qPCRs within 5min
**Nucleic acid extraction**		
Procedure and/or instrumentation	E	PowerSoil™ DNA Isolation kit, mechanical lysis
Name of kit and details of any modifications	E	PowerSoil™ DNA Isolation kit (MoBio Laboratories, Carlsbad, CA, USA), no modifications
Source of additional reagents used	D	DNA was eluted in DEPC-treated water
Details of DNase or RNase treatment	E	No treatment
Contamination assessment (DNA or RNA)	E	No template DNA controls included to DNA isolation, analyzed with qPCR
Nucleic acid quantification	E	Qubit® 2.0 Fluorometer
Instrument and method	E	Qubit® 2.0 Fluorometer (Thermo Fisher Scientific, Vienna, Austria)
Purity (A_{260}/A_{280})	D	
Yield	D	
RNA integrity method/instrument	E	Not relevant
RIN/RQI or Cq of 3' and 5' transcripts	E	Not relevant
Electrophoresis traces	D	Aliquots of qPCR products were analyzed by agarose gel electrophoresis
Inhibition testing (Cq dilutions, spike or other)	E	No inhibition determined (dissociation curves, gel electrophoresis analysis, specificity of the amplicons verified by DNA sequencing of the PCR products)
**Reverse transcription**		
Reaction conditions	E	Not relevant
Amount of RNA and reaction volume	E	Not relevant
Priming oligonucleotide and concentration	E	Not relevant
Reverse transcriptase and concentration	E	Not relevant
Temperature and time	E	Not relevant
Manufacturer of reagents and catalogue numbers	D	Not relevant
Cqs with and without RT	D	Not relevant
Storage conditions of cDNA	D	Not relevant
**qPCR target information**		
Gene symbol	E	18S rRNA gene
Sequence accession number	E	None known, see https://doi.org/10.1186/1471-2180-12-255
Location of amplicon	D	18S rRNA Gene, *S. cerevisiae* region 1199- 1549
Amplicon length	E	351bp
In silico specificity screen (blast, etc)	E	see https://doi.org/10.1186/1471-2180-12-255
Pseudogenes, retropseudogenes or other homologs?	D	Not relevant
Sequence alignment	D	see https://doi.org/10.1186/1471-2180-12-255
Secondary structure analysis of amplicon	D	
Location of each primer by exon or intron (if applicable)	E	Not relevant
What splice variants are targeted?	E	Not relevant
**qPCR oligonucleotides**		
Primer sequences	E	FungiQuant-F 5′-GGRAAACTCACCAGGTCCAG-3′
		FungiQuant-R 5′-GSWCTATCCCCAKCACGA-3′
RTPrimerdb identification number	D	Not determined, see https://doi.org/10.1186/1471-2180-12-255
Probe sequences	D	FungiQuant-probe FAM-5′-TGGTGCATGGCCGTT-3′-MGBEQ

Location and identity of any modifications	E	No modifications
Manufacturer of oligonucleotides	D	Eurofins (Vienna, Austria)
Purification method	D	HPLC
**qPCR protocol**		
Complete reaction conditions	E	See main manuscript
Reaction volume and amount of cDNA/DNA	E	25 µl reaction volume (incl. 5 µl DNA)
Primer, (probe), Mg++ and dNTP concentrations	E	See main manuscript
Polymerase identity and concentration	E	1.5U of Platinum® Taq DNA polymerase (Thermo Fisher Scientific, Vienna, Austria)
Buffer/kit identity and manufacturer	E	See main manuscript
Exact chemical constitution of the buffer	D	10×PCR Buffer, – Mg; (Invitrogen, Vienna, Austria)
Additives (SYBR green I, DMSO, etc.)	E	no further additives
Manufacturer of plates/tubes and catalog number	D	MicroAmp optical tube (0.2 µl; Applied Biosystems by Life Technologies)
Complete thermocycling parameters	E	94°C for 2 min and 45 cycles of 94°C for 30 s followed by 60 s at 60°C, melting curve 50°C to 90°C
Reaction setup (manual/robotic)	D	Manual
Manufacturer of qpcr instrument	E	Stratagene Mx3000P real-time PCR System (Agilent Technologies, Santa Clara, USA)
**qPCR validation**		
Evidence of optimisation (from gradients)	D	First, followed protocol in https://doi.org/10.1186/1471-2180-12-255, second, concentrations of primers in range of 200-400 nM, $MgCl_2$ ranging from 2 to 3.5 mM, as well as annealing/extension temperature, ranging from 60°C to 64°C were tested with new reagents used
Specificity (gel, sequence, melt, or digest)	E	Gel, sequence, melting curve
For SYBR green, Cq of the NTC	E	Not relevant, TaqMan assay
Standard curves with slope and y-intercept	E	Done
PCR efficiency calculated from slope	E	95.20%
Confidence interval for PCR efficiency or standard error	D	-
r^2 of standard curve	E	Between 0.997 and 1
Linear dynamic range	E	Determined, 6 log scales tested
Cq variation at lower limit	E	Less than 4% within replicates
Confidence intervals throughout range	D	
Evidence for limit of detection (LOD)	E	see https://doi.org/10.1186/1471-2180-12-255
If multiplex, efficiency and LOD of each assay.	E	Not relevant, no multiplexing
**Data analysis**		
qPCR analysis program (source, version)	E	Stratagene Mx3000P real-time PCR System (Agilent Technologies, Santa Clara, USA)
Method of Cq determination	E	Stratagene Mx3000P real-time PCR System settings (baseline subtracted curve fit, single threshold, automatically calculated). Threshold manually curated for maximum efficiency within linear range for each plate
Outlier identification and disposition	E	Done
Results of NTCs	E	No amplification
Justification of number and choice of reference genes	E	Not done
Description of normalisation method	E	Not done
Number and concordance of biological replicates	D	2 biological replicates
Number and stage (RT or qPCR) of technical replicates	E	2 technical replicates for all samples and standards
Repeatability (intra-assay variation)	E	Repeatable
Reproducibility (inter-assay variation, %CV)	D	Not determined (strongly recommended for clinical/diagnostic applications, but not other assays)
Power analysis	D	
Statistical methods for result significance	E	Wilcoxon Signed-Rank test
software (source, version)	E	R (version 3.2.5, psych package 1.6.12).
Cq or raw data submission using RDML	D	
