# Supplementary material for: Austrian Raw-Milk Hard-Cheese Ripening Involves Successional Dynamics of Non-Inoculated Bacteria and Fungi
Source: Foods. 2020 Dec 11;9(12):1851. doi: 10.3390/foods9121851 (PMC7763656; doi:10.3390/foods9121851)
Supplement: Supplementary file 1 [file foods-09-01851-s001.zip › Table_S2-MIQE_guidelines_dPCR.pdf]

Table S2. Minimum information for publication of digital PCR experiments (MIQE guidelines dPCR) for 18S rRNA gene dPCR.

Item to check	Importance	Remarks
**Experimental design**		**18S/FungiQuant Assay dPCR**
Definition of experimental and control groups	E	Two cheese diary facilities, A and B
Number within each group	E	Number of samples within each group (n=100), number of subgroups (n=5): ripening days 0; 14; 30; 90; 160; pooled samples
Assay carried out by core lab or investigator's lab?	D	Investigator's lab
Power analysis	D	
**Sample**		
Description.	E	DNA isolated from cheese rinds
Volume or mass of sample processed	E	250 mg pellet of the homogenized cheese rind sample in duplicate
Microdissection or macrodissection	E	Not relevant, environmental material
Processing procedure	E	DNA isolation using PowerSoil™ DNA Isolation kit
If frozen—how and how quickly?	E	Samples were stored on ice during transport to the laboratory and processed immediately. After DNA isolation, samples were frozen within 10 minutes at -80°C.
If fixed—with what, how quickly?	E	Samples were not fixed
Sample storage conditions and duration	E	Cheese rind samples were processed immediately. DNAs were stored 1-6 months at -80°C, after thawing on ice, DNA samples were pooled and applied to the dPCR.
**Nucleic acid extraction**		
Quantification—instrument/method	E	Qubit® 2.0 Fluorometer (Thermo Fisher Scientific, Vienna, Austria)
Storage conditions: temperature, concentration, duration, buffer	E	Temp. -80°C; DEPC
DNA or RNA quantification	E	DNA quantitication
Quality/integrity, instrument/method, e.g. RNA integrity/R quality index and trace or 3′:5′	E	
Template structural information	E	Genomic
Template modification (digestion, sonication, preamplification, etc.)	E	EcoRI; AluI
Template treatment (initial heating or chemical denaturation)	E	Without template treatment
Inhibition dilution or spike	E	Dilution
DNA contamination assessment of RNA sample	E	NTCs included to DNA isolation, analyzed with dPCR
Details of DNase treatment where performed	E	Without Dnase treatment
Manufacturer of reagents used and catalogue number	D	PowerSoil™ DNA Isolation kit (MoBio Laboratories, Carlsbad, CA, USA), no modifications
Storage of nucleic acid: temperature, concentration, duration, buffer	E	DNA was eluted in DEPC-treated water
**RT (If necessary)**		
cDNA priming method + concentration	E	Not relevant
One- or 2-step protocol	E	Not relevant
Amount of RNA used per reaction	E	Not relevant
Detailed reaction components and conditions	E	Not relevant
RT efficiency	D	Not relevant
Estimated copies measured with and without addition of RT	D	Not relevant
Manufacturer of reagents used and catalogue number.	D	Not relevant
Reaction volume (for 2-step RT reaction).	D	Not relevant
Storage of cDNA: temperature, concentration, duration, buffer.	D	Not relevant
**dPCR target information**		
Sequence accession number.	E	None known, see https://doi.org/10.1186/1471-2180-12-255
Amplicon location.	D	18S rRNA Gene, S. cerevisiae region 1199- 1549
Amplicon length.	E	351bp
In silico specificity screen (BLAST, etc.).	E	see https://doi.org/10.1186/1471-2180-12-255
Pseudogenes, retropseudogenes or other homologs?	D	Not relevant
Sequence alignment.	D	see https://doi.org/10.1186/1471-2180-12-255
Secondary structure analysis of amplicon and GC content	D	done
Location of each primer by exon or intron (if applicable)	E	Not relevant
Where appropriate, which splice variants are targeted?	E	Not relevant
**Item to check 2**	**Importance**	**Remarks**
**dPCR oligonucleotides**		
Primer sequences and/or amplicon context sequence[b]	E	FungiQuant-F 5′-GGRAAACTCACCAGGTCCAG-3′; FungiQuant-R 5′-GSWCTATCCCCAKCACGA-3′
RTPrimerDB (real-time PCR primer and probe database) identification number	D	See https://doi.org/10.1186/1471-2180-12-255
Probe sequences	D	FungiQuant-probe FAM-5′-TGGTGCATGGCCGTT-3′-MGBEQ
Location and identity of any modifications	E	No modifications
Manufacturer of oligonucleotides	D	Eurofins (Vienna, Austria)
Purification method	D	HPLC
**dPCR protocol**		
Complete reaction conditions	E	See main manuscript
Reaction volume and amount of RNA/cDNA/DNA	E	25 µl reaction volume (incl. 5 µl DNA)
Primer, (probe), Mg^{++} and dNTP concentrations	E	See main manuscript
Polymerase identity and concentration	E	AccuStart II Taq DNA Polymerase incl. to PerfeCTa Multiplex qPCR ToughMix (Quanta Biosciences)
Buffer/kit catalogue no. and manufacturer	E	PerfeCTa Multiplex qPCR ToughMix (Quanta Biosciences) # 95147-250; Fluorescein (VWR cat. Number)
Exact chemical constitution of the buffer	D	
Additives (SYBR green I, DMSO, etc.)	E	No further additives

Plates/tubes Catalogue No and manufacturer	D	Sapphire chips (Stilla Technologies)
Complete thermocycling parameters	E	95°C for 10 minutes, 45 cycles of 95°C for 10 sec and 60°C for 15 sec.
Reaction setup	D	See main manuscript
Gravimetric or volumetric dilutions (manual/robotic)	D	Manual
Total PCR reaction volume prepared	D	25µl
Partition number	E	25,000-30,000 per chamber
Individual partition volume	E	0.00058592 nL
Total volume of the partitions measured (effective reaction size).	E	25µl
Partition volume variance/SD	D	PerfeCTa mix: 5% (droplet diameter)
Comprehensive details and appropriate use of controls	E	Done
Manufacturer of dPCR instrument	E	NAICA™ SYSTEM for Crystal Digital PCR™ (Stilla Technologies)
**dPCR validation**		
Optimization data for the assay	D	Done at 60°C; the assay should be optimized by testing different hybridization temperatures.
Specificity (when measuring rare mutations, pathogen sequences etc.)	E	Not relevant
Limit of detection of calibration control	D	
If multiplexing, comparison with singleplex assays	E	Not multiplexing
**Data analysis**		
Mean copies per partition (λ or equivalent).	E	0.2 copies/ uL to 20,000 copies/ ul
dPCR analysis program (source, version).	E	Crystal Reader software (v2.1.6); Crystal Miner software (v2.1.6)
Outlier identification and disposition.	E	
Results of no-template controls.	E	Included, see Supplementary Figures
Examples of positive(s) and negative experimental results as supplemental data.	E	Included, see Supplementary Figures
Where appropriate, justification of number and choice of reference genes.	E	Not done, not relevant
Where appropriate, description of normalization method.	E	Not relevant
Number and concordance of biological replicates.	D	
Number and stage (RT or dPCR) of technical replicates.	E	
Repeatability (intraassay variation).	E	Done, repeatable
Reproducibility (interassay/user/lab etc. variation).	D	
Experimental variance or CI	E	CI 95%: all undigested samples <2.4%; for samples digested with EcoRI <3.32%; for samples digested with AluI >26.9%; NTC: inf%
Statistical methods used for analysis.	E	Microsoft Excel (2016)
Data submission using RDML (Real-time PCR Data Markup Language).	D	
