# Supplementary material for: Austrian Raw-Milk Hard-Cheese Ripening Involves Successional Dynamics of Non-Inoculated Bacteria and Fungi
Source: Foods. 2020 Dec 11;9(12):1851. doi: 10.3390/foods9121851 (PMC7763656; doi:10.3390/foods9121851)
Supplement: Supplementary file 1 [file foods-09-01851-s001.zip › Table_S3-Diversity.pdf]

Table S3. Species richness (Chao1), diversity (Shannon), evenness (Simpson) and diversity coverage (Good's coverage) estimators for bacterial and fungal 16S rRNA and ITS2 genes amplicon sequencing datasets, respectively.

Facility	Days of ripening	Bacteria				Fungi			
		Chao1	Shannon	Simpson	Good's coverage	Chao1	Shannon	Simpson	Good's coverage
A	0	24	2.87	0.72	1.00	14	0.81	0.25	1.00
	14	30	2.85	0.71	1.00	15	1.90	0.66	1.00
	30	32	3.40	0.79	1.00	16	2.75	0.80	1.00
	90	22	3.85	0.91	1.00	12	2.06	0.65	1.00
	160	37	3.96	0.89	1.00	16	2.52	0.75	1.00
B	0	43	3.12	0.78	1.00	34	2.19	0.71	0.99
	14	62	4.51	0.90	1.00	15	2.28	0.76	1.00
	30	25	3.50	0.85	1.00	14	2.48	0.75	1.00
	90	47	3.60	0.83	1.00	13	1.80	0.58	1.00
	160	31	3.16	0.78	1.00	11	1.55	0.58	1.00
