# Supplementary material for: Austrian Raw-Milk Hard-Cheese Ripening Involves Successional Dynamics of Non-Inoculated Bacteria and Fungi
Source: Foods. 2020 Dec 11;9(12):1851. doi: 10.3390/foods9121851 (PMC7763656; doi:10.3390/foods9121851)
Supplement: Supplementary file 1 [file foods-09-01851-s001.zip › Table_S4-FCE_qPCR.pdf]

Table S4. Fungal cell equivalents (FCEs) per 0.5 g rind cheese of two cheese production facilities (A and B) at day 0, 14, 30, 90, and 160 of ripening. [1]: IQR Interquartile range.

Facility	Days of ripening	Median	Min	Max	IQR [1]
A	0	1.46E+06	4.45E+05	1.52E+07	2.57E+06
	14	2.19E+06	1.50E+06	3.48E+07	1.51E+06
	30	6.18E+06	2.50E+06	3.02E+07	6.57E+06
	90	5.41E+06	3.17E+06	1.16E+07	2.31E+06
	160	5.63E+06	3.70E+06	6.22E+07	2.24E+06
B	0	4.21E+07	1.76E+07	1.32E+08	2.15E+07
	14	6.85E+07	7.07E+06	4.55E+08	5.86E+07
	30	9.08E+06	3.46E+06	1.24E+08	4.31E+07
	90	5.83E+07	8.25E+06	1.08E+08	6.34E+07
	160	1.26E+07	5.94E+06	5.87E+07	1.61E+07
