# Supplementary material for: Austrian Raw-Milk Hard-Cheese Ripening Involves Successional Dynamics of Non-Inoculated Bacteria and Fungi
Source: Foods. 2020 Dec 11;9(12):1851. doi: 10.3390/foods9121851 (PMC7763656; doi:10.3390/foods9121851)
Supplement: Supplementary file 1 [file foods-09-01851-s001.zip › Table_S5-FCE_qPCR_p-value.pdf]

Table S5. Statistically significant differences between different ripening times. Fungal cell equivalents (FCEs) per 0.5 g cheese rind (qPCR data) during ripening in two different cheese production facilities were used.

Facility	*p* -value				
	**0 vs.14**	**0 vs. 30**	**14 vs. 30**	**30 vs. 90**	**90 vs.160**
A	<0.001	<0.001	0.67	<0.001	0.19
B	<0.001	<0.001	0.21	<0.001	<0.001
