# Supplementary figures and images for: Austrian Raw-Milk Hard-Cheese Ripening Involves Successional Dynamics of Non-Inoculated Bacteria and Fungi
Source: Foods. 2020 Dec 11;9(12):1851. doi: 10.3390/foods9121851 (PMC7763656; doi:10.3390/foods9121851)

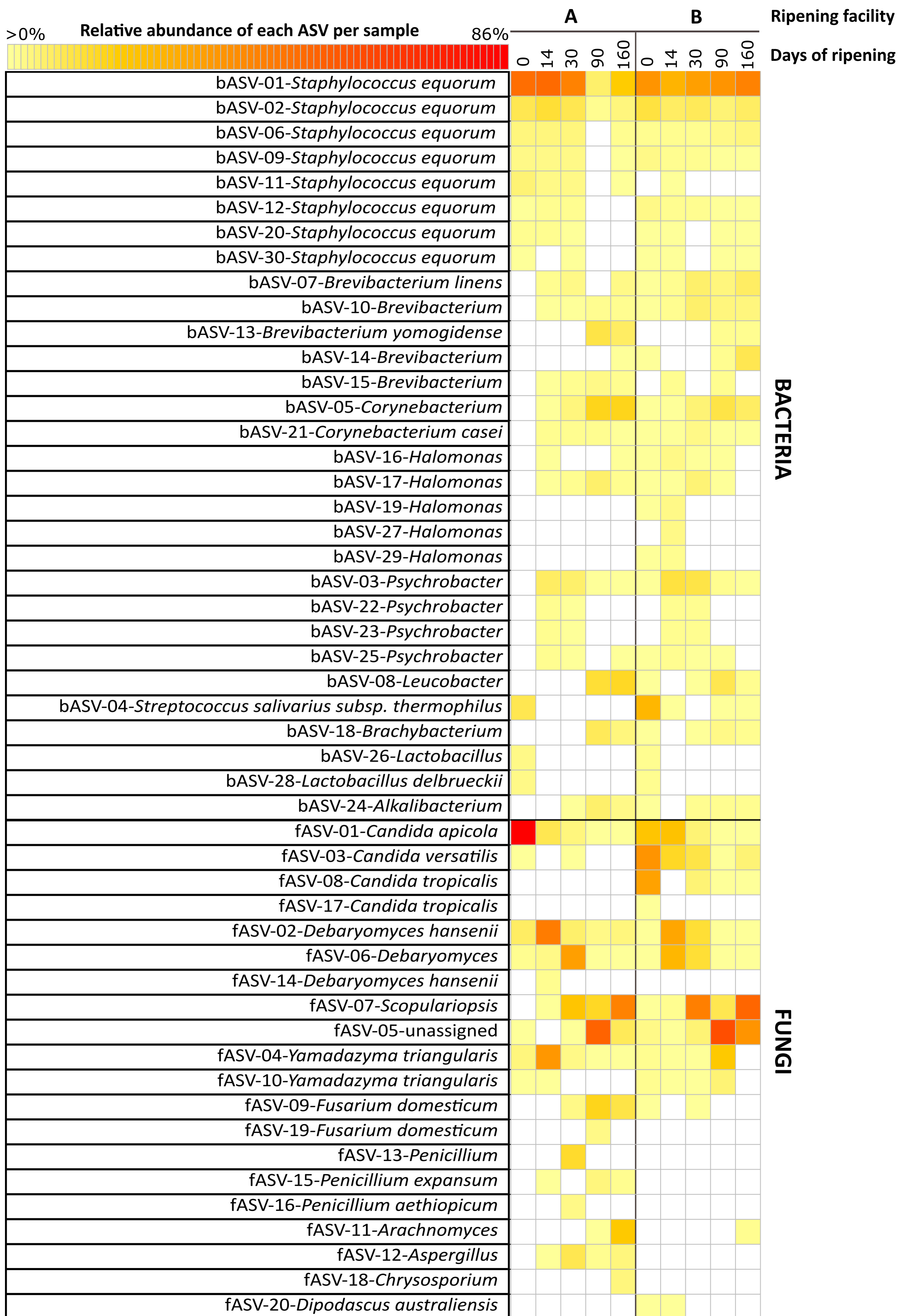

Supplement: Supplementary file 1 [file foods-09-01851-s001.zip › Figure_S3-heatmap_ASVs_relfreq.pdf]
